# Prenatal exposure to environmental contaminants and cord serum metabolite profiles in future immune-mediated diseases

**DOI:** 10.1038/s41370-024-00680-z

**Published:** 2024-04-27

**Authors:** Bagavathy Shanmugam Karthikeyan, Tuulia Hyötyläinen, Tannaz Ghaffarzadegan, Eric Triplett, Matej Orešič, Johnny Ludvigsson

**Affiliations:** 1 School of Science and Technology, Örebro University, SE-702 81 Örebro, Sweden; 2 School of Medical Sciences, Faculty of Medicine and Health, Örebro University, SE-702 81 Örebro, Sweden; 3 Department of Microbiology and Cell Science, Institute of Food and Agricultural Sciences University of Florida, Gainesville, 32611-0700 FL USA; 4 Turku Bioscience Centre, University of Turku and Åbo Akademi University, Turku, FI-20520 Finland; 5 Crown Princess Victoria’s Children’s Hospital and Division of Pediatrics, Department of Biomedical and Clinical Sciences, Linköping University, Linköping, SE-581 85 Sweden

**Keywords:** Autoimmune disease, environmental contaminants, exposome, lipidomics, metabolomics, type 1 diabetes

## Abstract

**Background:**

Prenatal exposure to environmental contaminants is a significant health concern because it has the potential to interfere with host metabolism, leading to adverse health effects in early childhood and later in life. Growing evidence suggests that genetic and environmental factors, as well as their interactions, play a significant role in the development of autoimmune diseases.

**Objective:**

In this study, we hypothesized that prenatal exposure to environmental contaminants impacts cord serum metabolome and contributes to the development of autoimmune diseases.

**Methods:**

We selected cord serum samples from All Babies in Southeast Sweden (ABIS) general population cohort, from infants who later developed one or more autoimmune-mediated and inflammatory diseases: celiac disease (CD), Crohn’s disease (IBD), hypothyroidism (HT), juvenile idiopathic arthritis (JIA), and type 1 diabetes (T1D) (all cases, *N* = 62), along with matched controls (*N* = 268). Using integrated exposomics and metabolomics mass spectrometry (MS) based platforms, we determined the levels of environmental contaminants and metabolites.

**Results:**

Differences in exposure levels were found between the controls and those who later developed various diseases. High contaminant exposure levels were associated with changes in metabolome, including amino acids and free fatty acids. Specifically, we identified marked associations between metabolite profiles and exposure levels of deoxynivalenol (DON), bisphenol S (BPS), and specific per- and polyfluorinated substances (PFAS).

**Impact statement:**

Abnormal metabolism is a common feature preceding several autoimmune and inflammatory diseases. However, few studies compared common and specific metabolic patterns preceding these diseases. Here we hypothesized that exposure to environmental contaminants impacts cord serum metabolome, which may contribute to the development of autoimmune diseases. We found differences in exposure levels between the controls and those who later developed various diseases, and importantly, on the metabolic changes associated with the exposures. High contaminant exposure levels were associated with specific changes in metabolome. Our study suggests that prenatal exposure to specific environmental contaminants alters the cord serum metabolomes, which, in turn, might increase the risk of various immune-mediated diseases.

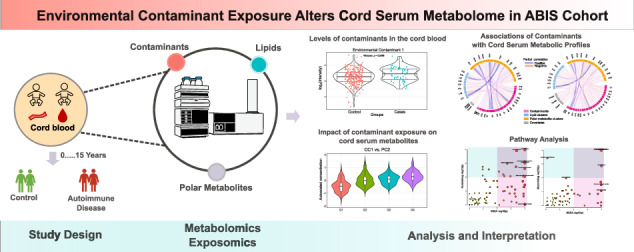

## Introduction

Exposure to environmental contaminants contributes to the global burden of many chronic diseases [1–5]. Over the past few decades, the prevalence of autoimmune diseases increased in both developed and developing countries, resulting in a high disease burden [6–10]. Autoimmune diseases are often manifested in early childhood and are also common among pregnant mothers [8, 11]. They are chronic, impact child growth and development, and require long-term management and care [12, 13]. Many studies suggest that a combination of genetic predisposition, environmental and maternal factors as well as their interactions play a significant role in the etiology of autoimmune diseases [14–21].

Exposure of humans to environmental chemicals begins already during the “sensitive window” of human early development, including the prenatal stage [22–25]. Prenatal exposure to PFAS and other contaminants has been associated with abnormal metabolism and progression to autoimmune diseases such as type 1 diabetes (T1D) [26], celiac disease (CD) [16, 18], Crohn’s disease (IBD) [27] later in life. PFAS exposure, for instance, alters the levels of phospholipids and contributes to the risk of T1D [26]. Although most autoimmune diseases share common pathogenicity and genetic risk factors [16, 28], their underlying pathogenic mechanisms are poorly understood. Besides exposure to environmental chemicals, perinatal factors such as low birth weight [29], the gut microbiome [30–35], and maternal diet [36–38] are also attributed to the progression of autoimmune diseases.

Given the potential impact of exposure to environmental contaminants and the role of maternal factors in the progression of autoimmune diseases [39], it is important to characterize the prenatal and early-life exposome to better understand the pathogenesis of autoimmune diseases. Herein, we hypothesized that exposure to environmental contaminants impacts cord serum metabolome, which may contribute to the development of one or more autoimmune diseases in the general population cohort (All Babies In Southeast Sweden, ABIS) [40, 41]. These outcomes were selected based on their incidence in the study cohort. It is well known that the impact of environmental exposures is highly individual, with strong gene-environment interactions factors playing a role in the health impacts of exposures. It is also well-known that several autoimmune diseases share common risk factors or pathogenic mechanisms. For instance, T1D and CD exhibit shared predisposing alleles in the class II HLA-region [28]. Around 6% of T1D patients also develop clinical CD, and individuals with CD face an increased risk of developing T1D before the age of 20 [42]. On the other hand, T1D, multiple sclerosis, and rheumatoid arthritis are categorized as T-cell-mediated autoimmune diseases [43]. Significantly, there are indications that fundamental processes governing T-cell functionality are interconnected with changes in cellular metabolic programs [44]. External perturbations to key metabolic processes can hinder T-cell activation, differentiation, and cytokine production.

In the ABIS cohort, we analyzed contaminants and metabolite profiles from cord serum collected at birth, using integrated exposomics and metabolomics approaches. We investigated (i) the levels of exposure and significant differences between controls and cases, (ii) the associations of contaminant exposure with cord serum metabolic profiles, and (iii) the impact of contaminant exposure levels on cord serum metabolic profiles.

## Materials and methods

### Study design

**Fig. 1:**
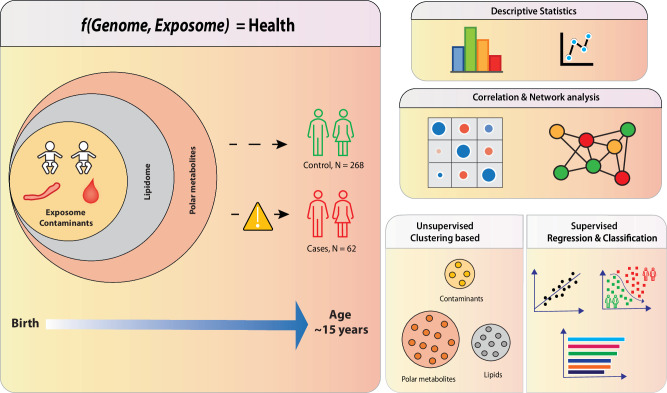
Summary of our work, which aimed to investigate how prenatal exposure to environmental contaminants alters the cord serum metabolome in the ABIS cohort. We used metabolomics to determine the levels of exposure to environmental contaminants and metabolites in the cord blood. Our work involved three main stages. Firstly, we examined the levels of exposure and significant differences between control (*N* = 268) and cases (*N* = 62). Secondly, we studied the associations of contaminant exposure with cord serum metabolic profiles. Finally, we investigated the impact of contaminant exposure on cord serum metabolites. Overall, our work sheds light on the effects of environmental contaminants on the cord serum metabolome, which may have implications for the future progression of autoimmune diseases.

ABIS, a general population cohort consisting of 17,000 children born 1st of Oct 1997–1st of Oct 1999, followed prospectively with regular follow-ups. ABIS is connected to the Swedish National Diagnosis Register which give information about diagnosis of autoimmune disease. Stool samples were collected from ca 1800 individuals at 1 year of age, and microbiome studies have been performed [34, 39]. The present study includes subjects (N = 62) from this group who later developed one or more autoimmune and inflammatory diseases such as CD (*N* = 28), IBD (*N* = 7), hypothyroidism (HT) (*N* = 6), juvenile idiopathic arthritis (JIA) (*N* = 9) and T1D (*N* = 12), along with their controls matched for sex and age at the time of diagnosis (*N* = 268). The average age of diagnosis was 11.5 years in CD, 16 years in IBD, 15 years in JIA, 16 years in HT, and 13.5 years in T1D group [39]. The cohort is representative of a general population in Sweden. Related to socioeconomical factors (*e.g*., parental education) or other lifestyle factors, there was no statistically significant differences between the groups. The cord blood samples collected during birth were subjected to metabolomics analysis. Figure 1 summarizes the study design and integrated workflow. For more detailed information about demographic characteristics and individual diseases, readers are referred to our previous study [39].

### Analysis of metabolome and environmental contaminants

A total of 330 cord blood samples were randomized and analyzed as described below. Shortly, two methods were applied for separate extraction of lipids and polar/semipolar metabolites and the extracts were then analyzed using an ultra-high-performance liquid chromatography quadrupole time-of-flight mass spectrometry (UHPLC-QTOFMS) as described previously [39] and the data were processed using MZmine 2.53 [45]. Quantification was performed using calibration curves and the identification was done with a custom database. Level 1 identification refers to identified compounds where reference compounds are available, while level 2 identification denotes compounds identified based on their MS/MS in comparison with mass spectrometry libraries, as defined by the Metabolomics Standards Initiative (MSI). Quality control was performed by analyzing pooled quality control samples. In addition, extracted blank samples, standards compounds, and reference plasma (NIST SRM 1950); purchased from the National Institute of Standards and Technology at the US Department of Commerce (Washington, DC, USA)), were analyzed as part of the quality control procedure.

#### Analysis of molecular lipids

10 µl of serum was mixed with 10 µl 0.9% NaCl and extracted with 120 µl of CHCl_3_: MeOH (2:1, v/v) solvent mixture containing internal standard mixture (*c* = 2.5 µg/ml; 1,2-diheptadecanoyl-sn-glycero-3-phosphoethanolamine (PE(17:0/17:0)), N-heptadecanoyl-D-erythro-sphingosylphosphorylcholine (SM(d18:1/17:0)), N-heptadecanoyl-D-erythro-sphingosine (Cer(d18:1/17:0)), 1,2-diheptadecanoyl-sn-glycero-3-phosphocholine (PC(17:0/17:0)), 1-heptadecanoyl-2-hydroxy-sn-glycero-3-phosphocholine (LPC(17:0)) and 1-palmitoyl-d31-2-oleoyl-sn-glycero-3-phosphocholine (PC(16:0/d31/18:1)) and, triheptadecanoylglycerol (TG(17:0/17:0/17:0)). The samples were vortexed and let stand on the ice for 30 min before centrifugation (9400 rcf, 3 min). 60 µl of the lower layer of was collected and diluted with 60 µl of CHCl_3_: MeOH. The samples were kept at -80 °C until analysis.

Samples were analyzed by UHPLC-QTOFMS (Agilent Technologies; Santa Clara, CA, USA). The analysis was carried out on an ACQUITY UPLC BEH C18 column (2.1 mm × 100 mm, particle size 1.7 μm) by Waters (Milford, USA). The eluent system consisted of (A) 10 mM NH_4_Ac in H_2_O and 0.1% formic acid and (B) 10 mM NH_4_Ac in acetonitrile (ACN): isopropanol (IPA) (1:1) and 0.1% formic acid. The gradient was as follows: 0–2 min, 35% solvent B; 2–7 min, 80% solvent B; 7–14 min 100% solvent B. The flow rate was 0.4 ml/min.

The following steps were applied in data processing with MZmine 2.53: (i) Mass detection with a noise level of 1000, (ii) Chromatogram builder with a minimum time span of 0.08 min, minimum height of 1000 and a m/z tolerance of 0.006 m/z or 10.0 ppm, (iii) Chromatogram deconvolution using the local minimum search algorithm with a 70% chromatographic threshold, 0.05 min minimum RT range, 5% minimum relative height, 1200 minimum absolute height, a minimum ration of peak top/edge of 1.2 and a peak duration range of 0.08–5.0, (iv), Isotopic peak grouper with a m/z tolerance of 5.0 ppm, RT tolerance of 0.05 min, maximum charge of 2 and with the most intense isotope set as the representative isotope, (v) Join aligner with a m/z tolerance of 0.009 or 10.0 ppm and a weight for of 2, a RT tolerance of 0.15 min and a weight of 1 and with no requirement of charge state or ID and no comparison of isotope pattern, (vi) Peak list row filter with a minimum of 10% of the samples (vii) Gap filling using the same RT and m/z range gap filler algorithm with an m/z tolerance of 0.009 m/z or 11.0 ppm, (vii) Identification of lipids using a custom database search with an m/z tolerance of 0.008 m/z or 8.0 ppm and a RT tolerance of 0.25 min. Identification of lipids was based on an in-house library based on LC-MS/MS data on retention time and mass spectra. The identification was done with a custom database, with identification levels 1 and 2, *i.e*., based on authentic standard compounds (level 1) or based on MS/MS identification (level 2).

Quantification of lipids was performed using a 7-point internal calibration curve (0.1–5 µg/mL) using the following lipid-class specific authentic standards: using 1-hexadecyl-2-(9Z-octadecenoyl)-sn-glycero-3-phosphocholine (PC(16:0e/18:1(9Z))), 1-(1Z-octadecenyl)-2-(9Z-octadecenoyl)-sn-glycero-3-phosphocholine (PC(18:0p/18:1(9Z))), 1-stearoyl-2-hydroxy-sn-glycero-3-phosphocholine (LPC(18:0)), 1-oleoyl-2-hydroxy-sn-glycero-3-phosphocholine (LPC(18:1)), 1-palmitoyl-2-oleoyl-sn-glycero-3-phosphoethanolamine (PE(16:0/18:1)), 1-(1Z-octadecenyl)-2-docosahexaenoyl-sn-glycero-3-phosphocholine (PC(18:0p/22:6)) and 1-stearoyl-2-linoleoyl-sn-glycerol (DG(18:0/18:2)), 1-(9Z-octadecenoyl)-sn-glycero-3-phosphoethanolamine (LPE(18:1)), N-(9Z-octadecenoyl)-sphinganine (Cer(d18:0/18:1(9Z))), 1-hexadecyl-2-(9Z-octadecenoyl)-sn-glycero-3-phosphoethanolamine (PE(16:0/18:1)) from Avanti Polar Lipids, 1-Palmitoyl-2-Hydroxy-sn-Glycero-3-Phosphatidylcholine (LPC(16:0)), 1,2,3 trihexadecanoalglycerol (TG(16:0/16:0/16:0)), 1,2,3-trioctadecanoylglycerol (TG(18:0/18:0/18:)) and 3β-hydroxy-5-cholestene-3-stearate (ChoE(18:0)), 3β-Hydroxy-5-cholestene-3-linoleate (ChoE(18:2)) from Larodan, were prepared to the following concentration levels: 100, 500, 1000, 1500, 2000 and 2500 ng/mL (in CHCl3:MeOH, 2:1, v/v) including 1250 ng/mL of each internal standard. For unknown lipids, the results are given as normalized peak areas, after normalization with the closest eluting internal standard.

#### Analysis of polar metabolites

40 µl of serum sample was mixed with 90 µl of cold MeOH/H2O (1:1, v/v) containing the internal standard mixture (Valine-d8, Glutamic acid-d5, Succinic acid-d4, Heptadecanoic acid, Lactic acid-d3, Citric acid-d4. 3-Hydroxybutyric acid-d4, Arginine-d7, Tryptophan-d5, Glutamine-d5, each at at c = 1 µgmL^−1^ and 1-D4-CA,1-D4-CDCA,1-D4-DCA,1-D4-GCA,1-D4-GCDCA,1-D4-GLCA,1-D4-GUDCA,1-D4-LCA,1-D4-TCA, 1-D4-UDCA, each at 0.2 1 µgmL^−1^) for protein precipitation. The tube was vortexed and ultrasonicated for 3 min, followed by centrifugation (10000 rpm, 5 min). After centrifuging, 90 µl of the upper layer of the solution was transferred to the LC vial and evaporated under the nitrogen gas to dryness. After drying, the sample was reconstituted into 60 µl of MeOH: H_2_O (70:30).

Analyses were performed on an Agilent 1290 Infinity LC system coupled with 6545 QTOFMS interfaced with a dual jet stream electrospray (dual ESI) ion source (Agilent Technologies; Santa Clara, CA, USA) was used for the analysis. Aliquots of 10 μL of samples were injected into the Acquity UPLC BEH C18 2.1 mm × 100 mm, 1.7-μm column (Waters Corporation, Wexford, Ireland), fitted with a C18 precolumn (Waters Corporation, Wexford, Ireland). The mobile phases consisted of (A) 2 mM NH_4_Ac in H_2_O: MeOH (7:3) and (B) 2 mM NH_4_Ac in MeOH. The flow rate was set at 0.4 mLmin^−1^ with the elution gradient as follows: 0-1.5 min, mobile phase B was increased from 5% to 30%; 1.5–4.5 min, mobile phase B increased to 70%; 4.5–7.5 min, mobile phase B increased to 100% and held for 5.5 min. A post-time of 5 min was used to regain the initial conditions for the next analysis. The total run time per sample was 20 min. The dual ESI ionization source settings were as follows: capillary voltage was 4.5 kV, nozzle voltage 1500 V, N2 pressure in the nebulized was 21 psi and the N2 flow rate and temperature as sheath gas was 11 Lmin^−1^ and 379 °C, respectively. In order to obtain accurate mass spectra in MS scan, the m/z range was set to 100-1700 in negative ion mode. MassHunter B.06.01 software (Agilent Technologies; Santa Clara, CA, USA) was used for all data acquisition.

MS data processing was performed using the same parameters as in lipidomic analysis.

Quantitation was done using 6-point calibration (PFOA *c* = 3.75–120 ng/mL, bile acids *c* = 20–640 ng/mL, polar metabolites *c* = 0.1 to 80 μg/mL). Quantification of other bile acids was done using the following compounds: chenodeoxycholic acid (CDCA), cholic acid (CA), deoxycholic acid (DCA), glycochenodeoxycholic acid (GCDCA), glycocholic acid (GCA), glycodehydrocholic acid (GDCA), glycodeoxycholic acid (GDCA), glycohyocholic acid (GHCA), glycohyodeoxycholic acid (GHDCA), glycolitocholic acid (GLCA), glycoursodeoxycholic acid (GUDCA), hyocholic acid (HCA), hyodeoxycholic acid (HDCA), litocholic acid (LCA), alpha-muricholic acid (αMCA), tauro-alpha-muricholic acid (T-α-MCA), tauro-beta-muricholic acid(T-β-MCA), taurochenodeoxycholic acid (TCDCA), taurocholic acid (TCA), taurodehydrocholic acid (THCA), taurodeoxycholic acid (TDCA), taurohyodeoxycholic acid (THDCA), taurolitocholic acid (TLCA), tauro-omega-muricholic acid (TωMCA) and tauroursodeoxycholic acid (TUDCA) and polar metabolites was done using alanine, citric acid, fumaric acid, glutamic acid, glycine, lactic acid, malic acid, 2-hydroxybutyric acid, 3-hydroxybutyric acid, linoleic acid, oleic acid, palmitic acid, stearic acid, cholesterol, fructose, glutamine, indole-3-propionic acid, isoleucine, leucine, proline, succinic acid, valine, asparagine, aspartic acid, arachidonic acid, glycerol-3-phosphate, lysine, methionine, ornithine, phenylalanine, serine and threonine. For other compounds detected, the results are given as normalized peak areas, after normalization with the closest eluting internal standard.

#### QC/QA

Quality control was accomplished both for lipidomics, polar metabolites and PFAS analysis by including blanks, pure standard samples, extracted standard samples, pooled quality control samples, and standard reference plasma samples (NIST SRM 1950). The pooled sample was prepared by taking an aliqout (10 µl) of each extract, separately for lipidomic and polar metabolite methods, then pooling them, and aliquoting the pool into separate vials. In lipidomic and metabolomic analyses, lipids that had >30% RSD in the pooled QC samples (an equal aliquot of each sample pooled together) or that were present at high concentrations in the extracted blank samples (ratio between samples to blanks < 5) were excluded from the data analyses.

### Statistical analysis

#### Data pre-processing and clustering

In this study, all data analyses were conducted using the R statistical programming language (version 4.1.2) (https://www.r-project.org/). The exposure datasets were pre-processed by log2 transformation and scaling to zero mean and unit variance (auto-scaled). For contaminant exposure analyses, individual contaminant and cluster-level analyses were performed. To cluster the contaminant data, we utilized the ‘*mclust*’ R package (version 5.4.10) for model-based clustering, selecting the model type and the number of clusters based on the highest Bayesian Information Criterion (BIC). To better understand the impact of exposure, we also incorporated lipidomics and metabolomics data from our previous study [39], which included eight lipid clusters (LCs) and twelve polar metabolite clusters (PCs), along with their individual features (Supplementary Table 1).

#### Demographic data and covariates

In terms of demographic data and covariates, the median age at the time of diagnosis for subjects who later developed autoimmune diseases was 15 years. We obtained information on birthweight, maternal age, gestational age, and BMI from the questionnaire. We have also data on lifestyle of the mothers and socioeconomic factors from both parents. We utilized birthweight and gestational age to calculate birthweight for gestational age (BWGA) Z-score, utilizing internationally validated infant growth charts developed by Fenton [46, 47].

#### Correlation and partial correlation analysis

Pairwise Spearman’s correlation between contaminants, lipid clusters (LCs), Polar metabolite clusters (PCs), and demographic variables (Z-score, Maternal age, BMI) was calculated and visualized using ‘*corrplot*’ R package (version 0.92). Two correlation plots were generated separately for control and cases. The correlation between variables visualised in the form of a matrix plot refers to positive and negative correlations and the strength of the association is referred to by the size of the dot or filled circles.

The Debiased Sparse Partial Correlation algorithm (DSPC) [48] was used to estimate partial correlation networks and visualized in the form of a chord diagram using ‘*circlize*’, R package (version 0.4.15) with edge ranges between ±0.14 to 1.0 and showing only correlations across contaminants, LCs, PCs, and demographic variables.

#### Univariate statistical analysis

To understand the impact of contaminant exposure levels on cord serum metabolome, the subjects were assigned to four quartiles based on the exposure levels. A two-way analysis of variance (ANOVA) test was performed followed by post-hoc Tukey’s test by using quartiles (Q1 to Q4) and subjects (cases and control) as factor variables. ANOVA test helps to identify any significant changes in the lipid or metabolite clusters and post-hoc Tukey’s test helps to identify the specific quartiles between which significant changes are observed.

#### Regression and classification analysis

Predictive logistic ridge regression (LRR) was performed to investigate the impact of individual contaminants on the stratification of autoimmune cases and controls. We have adapted the L2 regularization strategy to avoid multicollinearity among highly correlated predictors. Regularized regression modelling was performed using the ‘*glmnet*’ package in R (version 4.1-4). The hyper-parameter λ_minimum_ was determined by 10-fold cross-validation using the ‘*cv.glmnet*’ function from ‘*glmnet*’. The models were adjusted for Z-score, Maternal age and BMI. The accuracy of prediction was determined by AUCs, where the mean AUC of the model was estimated by bootstrapping, by resampling the exposure dataset into training (80%) and testing (20%) 10,000 times. All LRR models with a threshold of AUC > 0.60 were considered. Downsampling was performed to address the class imbalance problem (cases, n = 62, controls, n > 62). The ‘*caret*’ package (version 4.1.3) was used for the partition of data and the best models (based on mean AUCs) were assessed using Receiver Operating Characterisitic (ROC) curves using the ‘*ROCR*’ package. Additionally, we have performed a stepwise recursive feature elimination scheme to identify the minimum number of predictors that are needed to maximize the outcome.

To investigate the effect of contaminant exposure on the cord blood metabolome, we employed linear regression with L2 regularization (LR), using individual contaminant concentrations as predictors and the concentrations of significantly altered cord blood lipid or polar metabolites (and their cluster) as the response variable. Note that we conducted linear regression with L2 regularization for cases and controls together. The hyper-parameter λ_minimum_, which corresponds to the minimum cross-validation error, was selected through 10-fold cross-validation. We partitioned the data and performed resampling (10,000 iterations) as described earlier. The mean R square was used to estimate the accuracy of prediction and the significant impact of contaminant exposure on the cord blood metabolome.

Additionally, we determined the ranks of the predictors using LR and LRR modelling. For the LRR models, the ranks of the predictors were estimated based on the unit absolute differences in the odds ratio, while for the LR models, the ranks were based on the ridge coefficients normalized with the maximum value.

#### Pathway analysis

Pathway enrichment analysis comparing cases versus controls for Deoxynivalenol (DON) impact polar metabolites was performed using the MetaboAnalyst 5.0 web platform with the Functional Analysis (MS Peaks) module [49]. The input data for the pathway analysis consisted of complete high-resolution LC-MS spectral peak data obtained in negative ionization mode with a mass tolerance of 10 ppm. Linear regression analysis was performed to estimate the association between DON and polar metabolites while adjusting for Z-score, Maternal age, and BMI. The whole input peak list with FDR-corrected p-values and T-score was used for the pathway analysis. Overrepresented pathways were estimated against the background human scale metabolic model MNF (from MetaboAnalyst Mummichog package) and Kyoto Encyclopedia of Genes and Genomes (KEGG) pathways for *Homo sapiens* to determine the relative significance of the identified pathways [50]. The MetaboAnalyst 5.0 metabolomics pathway analysis (MetPA) tool [51] was used to calculate the Pathway Impact Scores [49, 52].

## Results

### Metabolomic analysis of the cord blood

Figure 1 summarizes the integration of exposomics and metabolomics workflows in the ABIS cohort. Cord serum samples were analyzed for a total of 545 lipids and 3417 polar metabolites, which were further grouped into 8 lipid clusters and 12 polar metabolite clusters, respectively. We previously found significant associations between the metabolite clusters and demographic variables or clinical parameters such as gestational age, maternal age, and birth weight [39]. To account for these associations, we used the Z-score as calculated from birth weight, gestational age, and maternal BMI as covariates in our analysis.

### Levels of contaminants in the cord blood

**Fig. 2:**
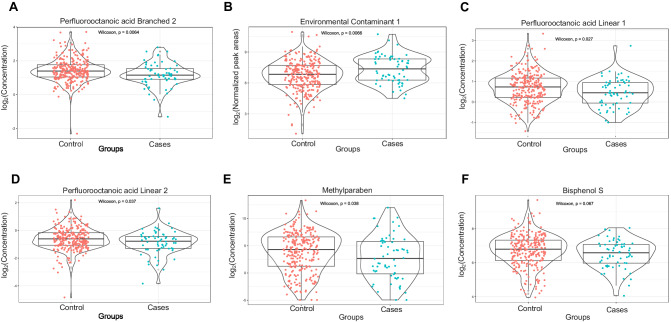
Box plots that illustrate the levels of selected contaminants in control and cases. The violin plots (**A**–**F**) on top of the box plots depict the distribution of the selected contaminants (log2 intensities). To test the mean difference between the control and cases, we conducted a Wilcoxon test. The p-values are provided to indicate the significance levels for the mean differences between the two groups for each contaminant (**A**–**F**). Specifically, *p* < 0.05 indicates statistical significance, and *p* < 0.1 suggests a trend toward significance. Overall, these results help to identify specific contaminants that may contribute to the altered cord serum metabolome in cases.

A total of 20 contaminants, including several PFAS compounds, were detected in cord blood samples from both control and case groups (Supplementary Table 2). Differences (*p* < 0.05) in concentration levels between control and case groups were observed for Perfluorooctanoic acid Branched 2, Environmental Contaminant 1, Perfluorooctanoic acid Linear 1, Perfluorooctanoic acid Linear 2 and Methylparaben (Fig. 2). The environmental contaminant 1 has been putatively identified as mOPFLCA *n* = 2, based on Norman suspect screening list, however, due to the lack of authentic standard, we were not able to verify the identity. At the individual disease level, Environmental Contaminant 1, Perfluorooctanoic acid Linear 2, and Perfluorooctanoic acid Branched 2 showed differences (*p* < 0.05) in concentration levels between control and individual disease groups (Supplementary Fig. 1). The contaminants were reduced to four clusters (CC1-CC4) consisting of eight contaminants, including Bisphenol S, Deoxynivalenol, Monobutyl phthalate, and a-Zearalanol in CC1; Ethylparaben, Methylparaben, and Propylparaben in CC2; Perfluorohexanesulfonic acid (PFHxS) and Perfluorohexanesulfonic acid Branched (PFHxSBr) in CC3; and seven PFAS and their fragments as part of CC4 (Supplementary Table 2). The clustering was guided by the Bayesian Information Criterion (BIC), selecting models with the highest BICs to evaluate performance and guide the clustering process.

### Exposure level of contaminants as a predictor for immune-mediated diseases

We employed predictive logistic ridge regression (LRR) models to stratify controls and cases based on their contaminant concentrations. The models were fitted using all predictors or by using the stepwise recursive feature elimination (RFE) method. The mean area under the curve (AUC) values for the models were 0.65 (95% CI 0.63-0.67) when using all predictors and 0.67 (95% CI 0.66-0.68) when using the stepwise RFE method (Supplementary Fig. 2). Our results showed that the contaminant concentration levels have a modest potential to differentiate controls from autoimmune diseases, as indicated by the mean AUC values (Supplementary Fig. 2). The ranks of individual contaminants (predictors) for separating controls and cases were estimated based on the unit absolute difference in odds ratios (Supplementary Fig. 2A).

### Associations between contaminants and cord serum metabolic profiles

**Fig. 3:**
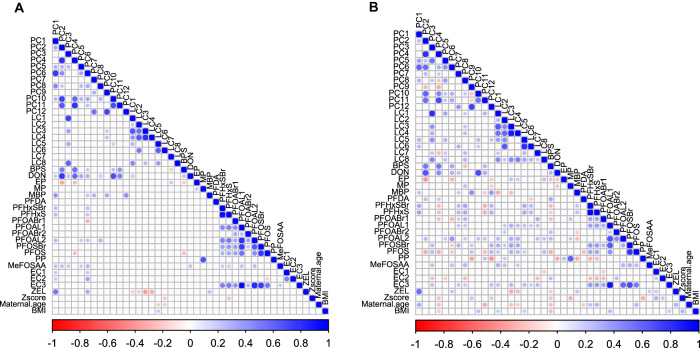
Correlations between contaminant exposure, metabolite clusters, and demographic data. Pairwise Spearman correlations were used to calculate the correlation coefficients between all cluster variables, contaminants, and demographic variables in the ABIS cohort separately for (**A**) controls and (**B**) cases. Positive and negative correlations are denoted by blue and red colours, respectively. The size of the dot in each cell corresponds to the strength of the pairwise correlation. To improve visualization, we only show correlations between +/- 0.20 to 1.0 in the plots. Overall, these correlation plots provide a comprehensive overview of the complex relationships between environmental contaminants, metabolites, and demographic factors in the ABIS cohort.

**Fig. 4:**
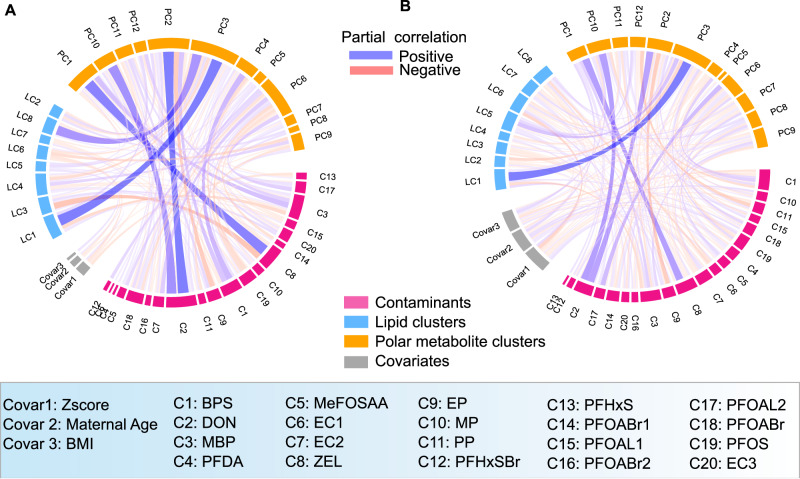
Partial correlation networks swhowing associations between contaminant exposure, metabolite clusters, and demographic data. The networks are shown separately for (**A**) controls and (**B**) cases. To filter out spurious or indirect correlations between variables, we used the Debiased Sparse Partial Correlation (DSPC) algorithm [48] to only show direct correlations. We used a conservative cut-off between +/− 0.14 to 1.0 to visualize the correlations and project only correlations across groups (Contaminants, metabolite clusters, and covariates/demographic data). Positive and negative correlations are denoted by blue and red lines, respectively. Overall, this partial correlation network provides a more detailed view of the complex relationships between environmental contaminants, metabolites, and demographic factors in the ABIS cohort.

We found significant associations between contaminants and cord serum metabolic profiles (Fig. 3). Specifically, more associations were observed between PFAS exposures and metabolic profiles in cases than in controls. Maternal age was positively associated with metabolite cluster PC1 in cases but not in controls (Fig. 3). We also performed partial correlation network analysis to identify non-spurious associations and Fig. 4 shows the marked associations between contaminants and cord serum metabolic profiles along with demographic variables. Notably, the controls exhibit stronger associations between contaminants and cord serum metabolic profiles compared to cases (Fig. 4). In the case group (Fig. 4B), the covariates Z-score, maternal age, and BMI showed a stronger association with exposure and cord serum metabolic profiles compared to the control group (Fig. 4A). The mycotoxins including deoxynivalenol were found to be associated with PC2 (phosphatidylcholines) and PC10 (unknowns), while a-zearalanol was associated with PC1 (lysophosphatidylcholines, sphingomyelins, and ceramides) (Fig. 4).

### Impact of contaminant exposure on cord serum metabolites associated with immune-mediated diseases

**Fig. 5:**
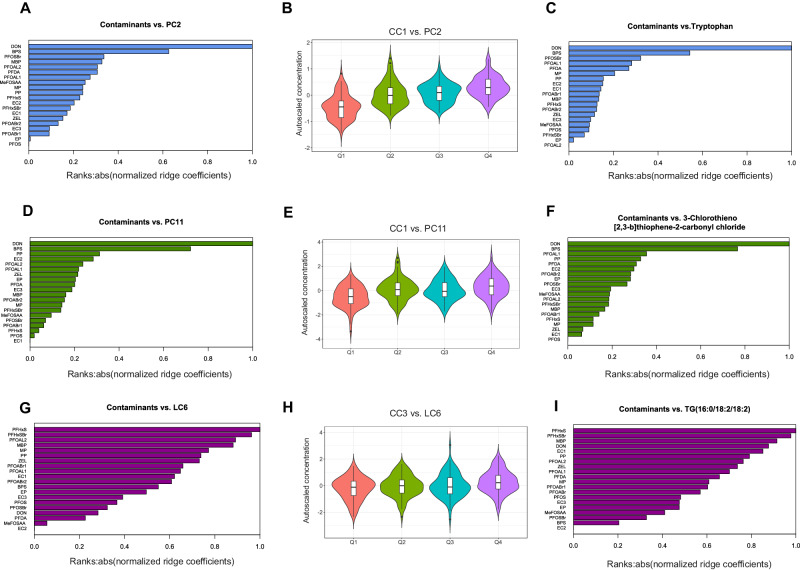
Impact of environmental contaminants on cord serum metabolites. Horizontal bar plots (**A**, **D**, **G**) and (**C**, **F**, **I**) display the ranks of contaminants as predictors of metabolite clusters (PC2, PC11, and LC6) and individual metabolites (Tryptophan, 3-Chlorothieno [2,3-b]thiophene-2-carbonyl chloride, and TG(16:0/18:2/18:2)), respectively. The potential impact of contaminants on metabolite clusters or individual metabolites is determined by their rank at the top of the bar plot. The ranks are based on their absolute normalized (ridge) regression coefficients. Violin plots (**B**, **E**, **H**) show the levels of metabolites (clusters) associated with levels of exposure to contaminants from contaminants clusters 1 (CC1) and 3 (CC3). The violin plot represents the density of the sample within each quartile, and their distribution is represented using a box plot at the centre. Two-way ANOVA followed by post-hoc Tukey´s HSD test was used to compare the mean difference between levels of metabolites (along quartiles).

The samples were stratified into quartiles based on their level of exposure to contaminants, and the impact of exposure on metabolite levels was assessed at both individual contaminant levels and cluster levels (CC1-CC4) (Supplementary Tables 3–6). The polar metabolite clusters displayed more significant mean differences between the highest (Q4) and lowest (Q1) quartiles, as shown in Supplementary Tables 5,6. In CC1, significant mean differences between Q4 and Q1 were observed for LC3, LC4, and LC7 at the lipid cluster level (Supplementary Table 4). In CC3, which includes perfluorohexanesulfonic acid (PFHxS) and branched (PFHxSBr), significant mean differences between the highest and lowest quartiles were observed for LC5 and LC6 (Supplementary Table 4 and Fig. 5H).

Linear ridge regression (LR) was performed to determine the quantitative effect of contaminant concentration levels on cord serum metabolic profiles. The results showed that polar metabolites in cord serum were more highly impacted by contaminant exposure than lipid levels. Specifically, six polar metabolite clusters, PC2 (R^2^ = 0.72), PC6 (R^2^ = 0.53), PC4 (R^2^ = 0.52), PC1 (R^2^ = 0.48), PC10 (R^2^ = 0.48), and PC11 (R^2^ = 0.32), showed significant associations with exposure levels (Fig. 5 and Fig. S3). At the individual metabolite level, amino acids such as tryptophan (Fig. 5C), Serine of PC2, and 3-Chlorothieno [2,3-b]thiophene-2-carbonyl chloride of PC11 showed a significant impact (Fig. 5F). According to the ranks of the predictors (contaminants), deoxynivalenol (DON) and Bisphenol S were the top linear predictors of cord serum metabolites and clusters PC2 and PC11 (Fig. 5A–F).

Although the contaminants from clusters CC1 and CC3 showed a significant association between quartiles (Q4 vs. Q1) and lipid cluster levels LC3, LC4, LC5, LC6, and LC7, their strength of association based on LR models was comparatively weaker (Fig. 5G–I and Supplementary Fig. 3). For example, the lipid cluster LC6, which mainly comprises triglycerides containing monounsaturated fatty acid (MUFA) and polyunsaturated fatty acids (PUFA), showed a weaker association (R^2^ = 0.04) with contaminant exposures (Fig. 5G–I).

### Pathway analysis of deoxynivalenol exposure

Metabolic pathway enrichment analysis was performed to evaluate the impact of DON on polar metabolites in both control and case groups separately. DON was found to be the top predictor that impacted polar metabolite clusters PC2, PC4, PC10, and PC11, as shown in Fig. 5 and Supplementary Fig. 3. Both Mummichog and GeneSet Enrichment Analysis (GSEA) algorithms were utilized using MetaboAnalyst 5.0 [49, 50]. Based on the pathways identified by the impact of DON exposure, both control and case groups showed common and specific metabolic pathways, as presented in Supplementary Tables 7–10.

**Fig. 6:**
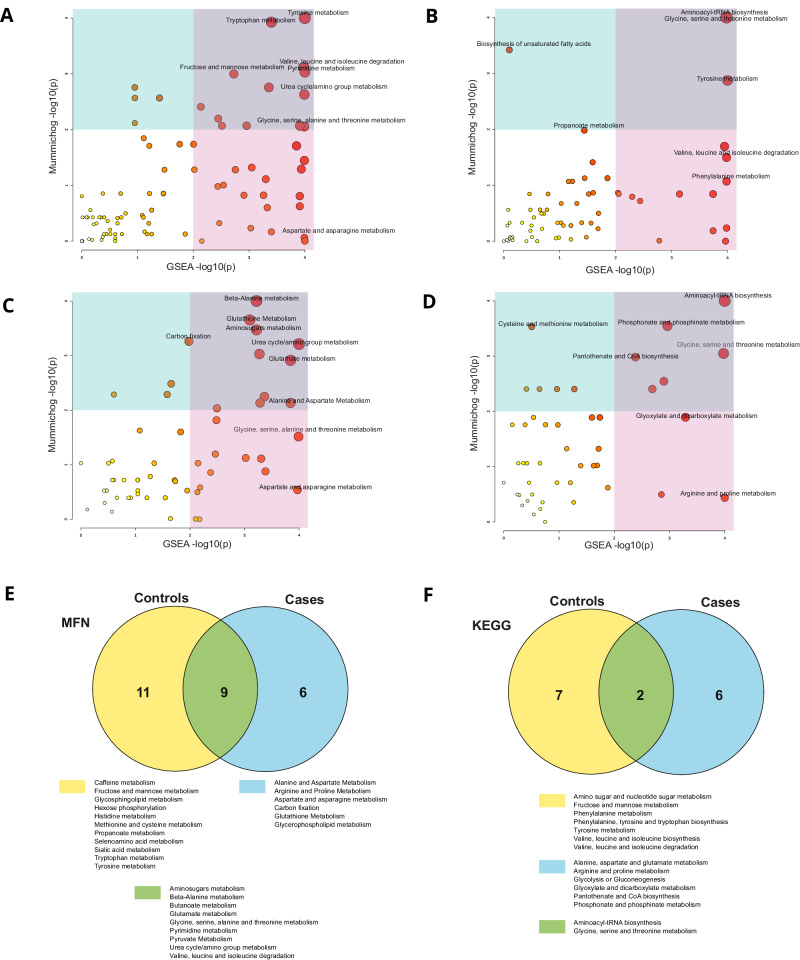
Pathway enrichment analysis comparing cases versus controls for Deoxynivalenol (DON) impact polar metabolites. The scatter plots depict the p-values using two different pathway maps: MFN pathway maps on the left panels and KEGG pathway maps on the right panels. The pathway analysis methods Mummichog and GSEA are used on the y-axis and x-axis, respectively, for both control (**A**, **B**) and cases (**C**, **D**). The size of the circle on each scatter plot represents the pathway impact value. The Venn diagram shows the common and unique pathways in controls and cases (**E**, **F**). For more detailed information, such as the number of metabolites in the pathways (total number/hits/significant hits) and p-values, please refer to the supplementary information.

The MFN pathway map revealed that DON exposure was associated with ‘Tyrosine and Tryptophan metabolism’ in the control group but not in cases. Also ‘Glutathione Metabolism’, ‘Alanine and Aspartate Metabolism’, and ‘Glycerophospholipid metabolism’ were found to be associated with exposure to DON in cases, but not in controls (Fig. 6A, C and E; Supplementary Tables 7 and 9). Similarly, based on the KEGG pathway maps, ‘Aminoacyl-tRNA biosynthesis’ and ‘Glycine, serine, and threonine metabolism’ were common among control and case groups, while several other metabolic pathways were specific to each group (Fig. 6B, D, and F, Supplementary Tables 8 and 10). In summary, the pathway enrichment analysis provided insights into the metabolic pathways affected by DON exposure in both control and case groups. The results highlight the differences in the impacted pathways between the two groups based on the exposure to DON.

## Discussion

We performed integrated exposomics and metabolomics to detect the levels of exposure to contaminants and metabolite levels in cord serum. This comprehensive approach allowed us to assess the individual associations of environmental exposures and metabolic profiles on autoimmune diseases in the ABIS cohort. In our previous study we found similarities in metabolic profiles across different autoimmune diseases at birth [39]. In order to avoid class imbalance problems, here we pooled all individual diseases together. We detected 20 contaminants, encompassing several PFAS compounds, Bisphenol S, and mycotoxins like Deoxynivalenol (DON), in cord blood samples from both control and case groups. Previous studies, including our own, have reported detectable levels of PFAS compounds [18, 26], Bisphenol S exposure [53], and the presence of mycotoxins, including Deoxynivalenol (DON), in cord blood samples [54]. These findings provide a backdrop for our investigation into the associations between these contaminants and autoimmune diseases in the ABIS cohort. We were able to demonstrate significant differences in the exposure levels of certain contaminants, such as Perfluorooctanoic acid Branched 2, Environmental Contaminant 1, Perfluorooctanoic acid Linear 1, Perfluorooctanoic acid Linear 2, and Methylparaben, in cord blood between the control and case groups. However, it is important to note that while these differences were statistically significant, the effect sizes were relatively modest. This suggests that while contaminants do play a role in distinguishing between controls and autoimmune diseases, they are unlikely to be the sole risk factors. Various factors including genetics, environmental triggers, and lifestyle factors, and their mutual interactions, contribute to the development of autoimmune diseases [14–21, 55].

Our study revealed differences in exposure and metabolite profiles between individuals who later developed autoimmune diseases and controls, particularly in relation to Z-score, mothers’ age, and BMI. This suggests that there may be differences in maternal factors between the two groups even at birth. We also observed that high levels of exposure to environmental contaminants were associated with changes in amino acid and free fatty acid profiles in the cord blood metabolome. Although we previously found a significant impact on lipid profiles, particularly triacylglycerols, the strength of association is weaker compared to the effect on polar metabolites [39]. Among the 20 contaminants measured in our study, DON, Bisphenol S, and some branched PFAS compounds are the primary predictors of changes in cord serum metabolic profiles. While the associations between PFAS exposure and their marked effect on metabolism leading to autoimmune diseases have been well documented in previous studies [18, 26, 56, 57], the exposure to DON and BPS and their impact on autoimmune diseases is less studied.

While our study detected Bisphenol S (BPS) and not Bisphenol A (BPA) due to this compound being present also in our blank samples, it’s noteworthy that BPA, a common chemical found in plastics, has been associated with alterations in amino acid metabolism [58]. BPA has been linked to changes in phenylalanine, tryptophan, tyrosine, lysine, and arginine metabolism, with a particular impact on female infants [59]. In the case of BPS, it was shown to have sex- and diet-dependent effects on the development of type 1 diabetes (T1D) in non-obese diabetic (NOD) mice. Female mice exposed to BPS on a soy-based diet exhibited delayed T1D development, while males showed increased insulin resistance [60]. These findings suggest that both BPA and BPS can influence metabolism and immune responses, potentially contributing to autoimmune diseases like T1D, although there is less evidence regarding the effect of BPS in humans.

Deoxynivalenol (DON) exposure in pregnant women has been reported in various studies. In the UK, pregnant women from diverse backgrounds showed detectable urinary DON levels, with South Asian women having higher exposure, primarily from bread consumption [61]. Similarly, in Norway, DON, a common mycotoxin in cereals, was found in various cereal-based foods, potentially affecting the immune system, particularly in infants and young children [62]. In pregnant Egyptian women, DON co-occurred with other mycotoxins, raising concerns about maternal and fetal health [63].

These findings emphasize the importance of assessing DON exposure in pregnant women and its potential health implications. DON exposure, prevalent in grains, adversely affects the immune system in both humans and animals and has been linked to alterations in gut microbiota [64]. This immunotoxicity induced by DON involves mechanisms such as MAPK activation, ER stress, and mitochondrial signaling pathways [64].

To study further the potential mechanisms underlying these associations, we conducted a pathway analysis of DON exposure on polar metabolites within both control and case groups. This analysis revealed that DON had distinct impacts on metabolic pathways in these groups. In the control group, DON exposure was associated with alterations in multiple amino acid metabolic pathways, as well as with fructose and mannose metabolism and urea cycle. Amino acids are also linked with immune cell functions, as they are key nutrients for immune cells. Alteration of tryptophan metabolism has been linked with inflammatory and immune responses, with tryptophan catabolism being recognized as an important player in inflammation and immune response [65, 66]. Our results also agree with studies in animal models, which have demonstrated changes in tryptophan metabolism following exposure to DON [67]. In addition, low circulating concentrations of tryptophan have been observed in infectious and autoimmune diseases and also in disorders that involve cellular (Th1-type) immune activation [68]. We have also earlier shown in the same cohorts as studied here that autoimmunity was associated with tryptophan metabolism [39]. Also in the case group, we observed associations in multiple amino acid metabolic pathways, urea cycle, and sugar metabolism as well as on other pathways that suggests disruptions in antioxidant defense systems and lipid metabolism.

Of the affected pathways, glutathione has been suggested to be a primer for T-cell metabolism for inflammation [69] while T-cell activation has shown to be dependent on extracellular alanine [70]. Also, glycerophospholipid metabolism has been indicated to play a significant role in systemic immune and low-grade inflammatory states [71].

These findings align with previous research indicating that DON can induce oxidative stress by reducing antioxidant enzyme activity and enhancing lipid peroxidation [72]. The marked association between DON and phosphatidylcholines in our study suggests a potential link between mycotoxin exposure and alterations in lipid metabolism, particularly in the context of phosphatidylcholines. This finding is noteworthy, as specific phosphatidylcholines were previously identified as persistently down-regulated in children who later progressed to islet autoimmunity [37] and clinical T1D [15]. Thus, the oxidative stress response and its impact on lipid metabolism, triggered by DON exposure, may play a pivotal role in the pathogenesis of autoimmune diseases, warranting further investigation.

These differential effects of DON exposure on metabolic pathways between control and case groups highlight the intricate relationship between environmental exposures, metabolism, and immune dysregulation in the context of autoimmune diseases. The difference between the two groups also suggest a complex gene-environment interaction that could be underlying the observed changes in responses. While our study contributes to our understanding of the metabolic consequences of DON exposure, it’s essential to consider these findings within the broader context of various factors, including genetics, environmental triggers, and lifestyle factors, which collectively contribute to the development of autoimmune diseases. Understanding these effects is crucial when assessing DON exposure during pregnancy and its potential health consequences. In this study, the median age of diagnosis of autoimmune diseases was higher compared to previous studies in genetically high-risk cohorts [14–16]. Despite some common metabolic patterns [39], there were differences and limitations to consider. One important limitation of our study was the small sample size within each disease group, which restricted our analysis. Another limitation of our study was the lack of maternal exposure and longitudinal exposure data at different time points between birth and the onset of autoimmune diseases, which could explain their age-dependent progression.

Our previous studies in T1D [26] and CD [18] cohorts have mainly focused on the associations of PFAS exposure with the disease risk. Here we detected the levels of other contaminants such as Bisphenol S and some mycotoxins including DON and a-Zearalanol, which potentially show the differences in exposures. Mycotoxins are common contaminants of cereals and grains, and exposure to them is also associated with autoimmune disorders [64, 73–75]. This emphasizes the need for caution and control over mycotoxin exposure, particularly during pregnancy and critical developmental stages.

Altogether, our results show that high prenatal exposure to environmental contaminants associated with altered cord serum metabolite levels and may result in the progression of autoimmune diseases in the ABIS cohort. Other factors such as Z-score, maternal age, and BMI are associated with contaminant exposure levels. Mechanistic studies are required to elucidate pathways of disease progression upon exposure.

### Supplementary information

Supplementary Material

## Data Availability

Data are available upon reasonable request after ethical approval and an appropriate institutional collaboration agreement. These data are not available to access in a repository owing to concern that the identity of patients might be revealed inadvertently.
