# Supplementary material for: Prenatal exposure to environmental contaminants and cord serum metabolite profiles in future immune-mediated diseases
Source: J Expo Sci Environ Epidemiol. 2024 Apr 27;34(4):647–58. doi: 10.1038/s41370-024-00680-z (PMC11303251; doi:10.1038/s41370-024-00680-z)
Supplement: Supplementary file 1 — Supplementary Material [file 41370_2024_680_MOESM1_ESM.pdf]

Prenatal exposure to environmental contaminants and cord serum metabolite profiles in future immune-mediated diseases

Supplementary Material

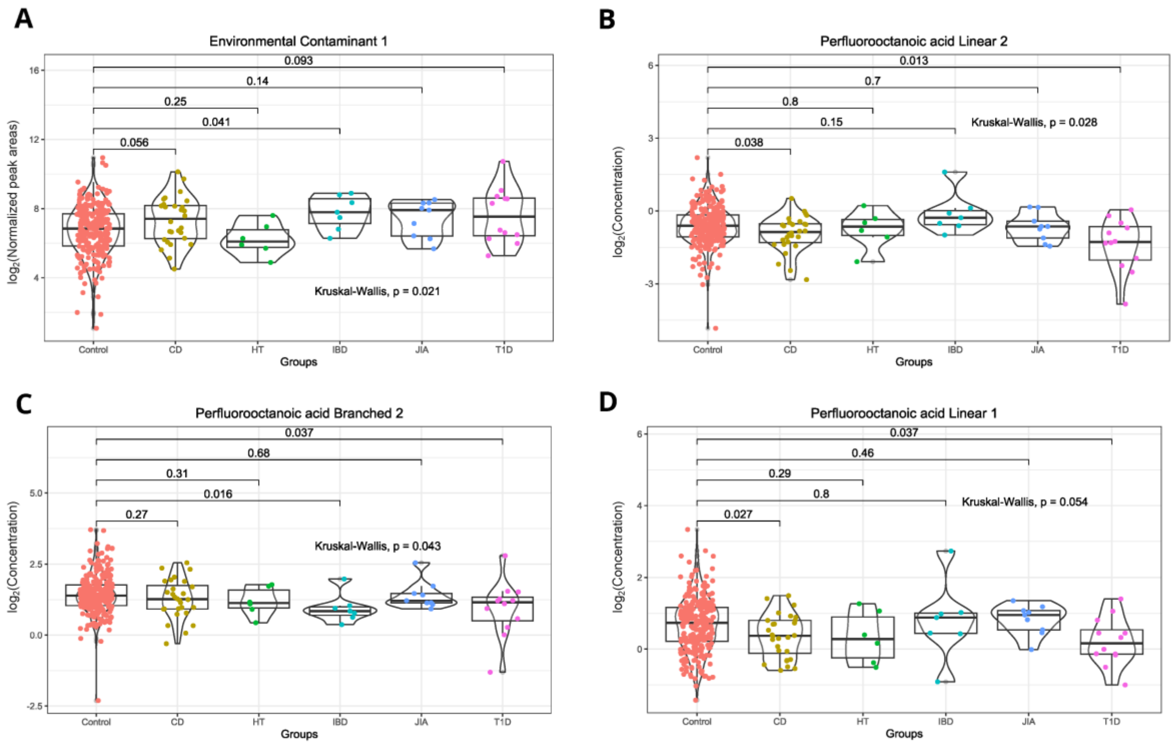

Supplementary Fig. 1. Box plots that show the levels of selected contaminants in the ABIS cohort at the individual disease levels. The violin plots (A-D) on top of the box plots illustrate the distribution of the selected contaminants (log2 intensities). To compare multiple group means, we used the Kruskal-Wallis Test, and for pairwise comparison against the reference (Control), we used the Wilcoxon Test. The p-values are provided to indicate the significance levels for the mean differences between the two groups (control vs. cases) for each contaminant (A-D). Specifically, $p < 0.05$ indicates statistical significance, and $p < 0.1$ suggests a trend toward significance. CD, HT, IBD, JIA, and T1D refer to Celiac disease, Hypothyroidism, Crohn's disease, Juvenile Idiopathic Arthritis and Type 1 Diabetes respectively.

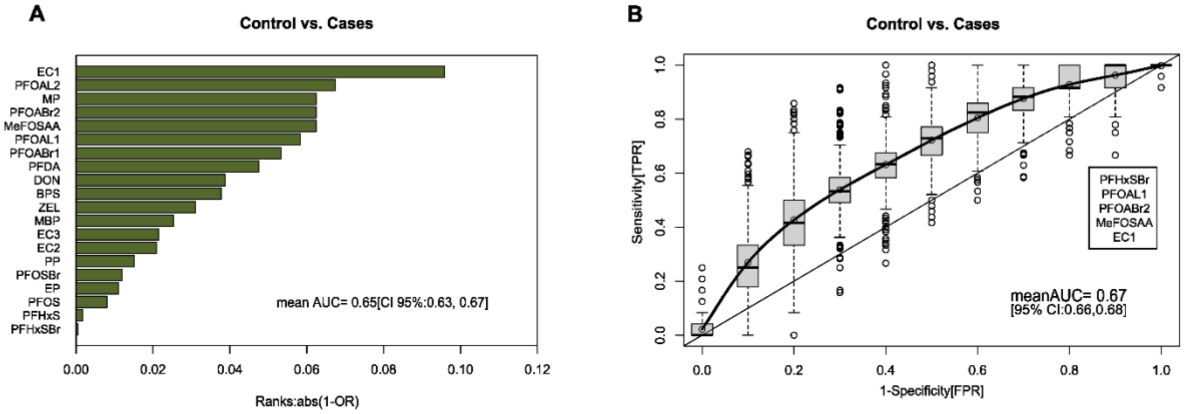

Supplementary Fig. 2. Classification of controls and immune-mediated diseases using contaminant exposure as predictors. In panel A, the ranks of the predictors (contaminants) obtained from the Logistic ridge regression (LRR) model, adjusted by Z-Score, Maternal age and BMI, are presented. The greatest contributing contaminants (predictors) that aided in the classification of control vs. cases (mean AUC = 0.65, 95% CI: 0.63–0.67) are shown at the top of the chart. In panel B, the Receiver Operating Characteristic (ROC) and AUC values from stepwise-predictive LRR models (10-fold cross-validation) are shown. An optimal set of five contaminants (predictors) (AUC = 0.67, 95% CI: 0.66–0.68) associated with the classification of control vs. cases are presented.

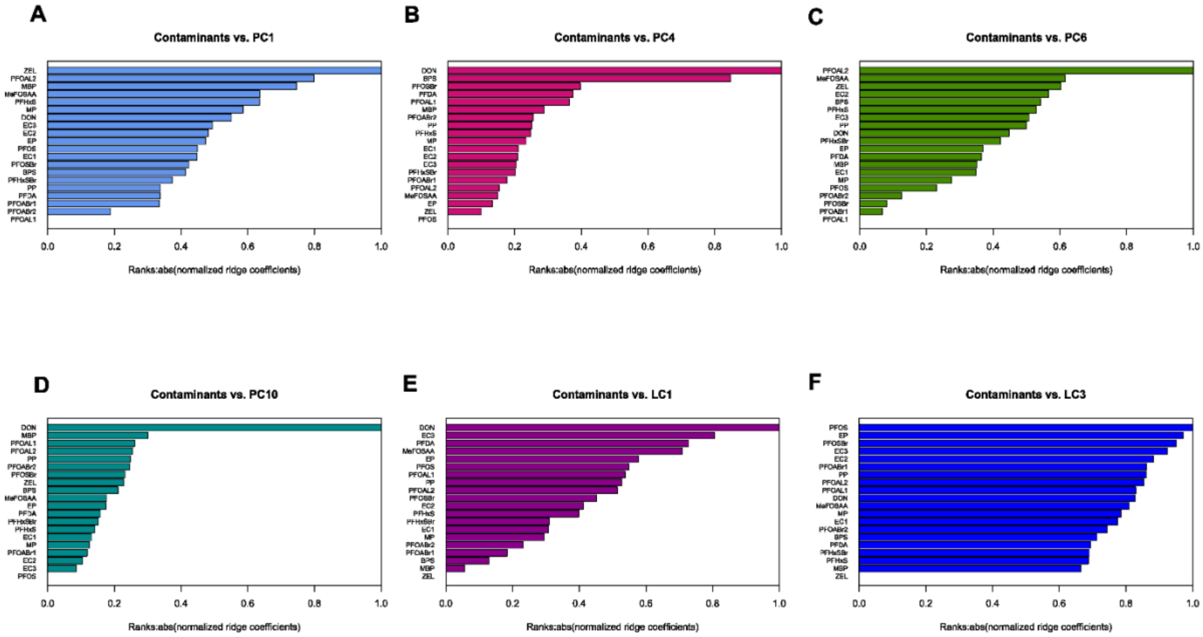

Supplementary Fig. 3. Association between exposure to environmental contaminants and the alteration of cord serum metabolites. The bar plots (A-F) show the linear predictors of changes in cord serum metabolite profiles, which are analysed in the form of metabolite clusters (Polar metabolite and lipid clusters). At the top of each bar plot (A-F), the most contributing contaminants (predictors) associated with selected metabolite clusters are shown. The ranks of the predictors are based on their absolute normalized (ridge) regression coefficients. The bar plots (A-D) represent the contaminant exposure as linear predictors of changes in polar metabolite clusters PC1, PC4, PC6 and PC10, while the bar plots (E-F) represent the contaminant exposure as linear predictors of changes in lipid clusters LC1 and LC3.

Supplementary Table 1. Description of lipid (LC) and polar metabolite (PC) clusters.

Cluster	Main classes of compounds	Specific examples
LC1	LPC, SM, Cer	SM(42:3), LPC(22:5), Cer(d18:1/24:0)
LC2	PC, PC_O	PC(40:8), PC(40:6), PC(O-40:4)
LC3	CE, Lac/HexCer, PC, PI, SM	CE(18:0), CE(18:1), Hexcer(d18:1/24:0)
LC4	PC_PUFA, LPC_PUFA	LPC(18:2), LPC(20:4), PC(38:4)
LC5	TG_SFA	TG(14:0/16:0/18:1),TG(16:0/16:0/16:0), TG(50:0)
LC6	TG_MUFA, TG_PUFA	TG(58:9), TG(18:1/18:1/22:6), TG(58:6)
LC7	Unknowns	Putative identifications: TGs
LC8	Unknowns	Putative identifications: various phospholipids
PC1	Bile acids, microbial metabolites	CA, CDCA, GCA, 3-indoleacetic acid
PC2	Amino acids	Valine, Phenylalanine, lysine, serine
PC3	Free fatty acids, lipids	Arachidonic acid, 16-Hydroxypalmitate, LPC(17:0)
PC4	Unknowns, highly polar compounds	
PC5	Free fatty acids, lipids	C16:1, C18:2, linoleic acid
PC6	Unknowns	
PC7	Unknowns	
PC8	Unknowns	
PC9	Unknowns	
PC10	Unknowns	
PC11	Unknowns, highly polar compouds	Putative identifications: exogeneous compounds
PC12	Unknowns	

Supplementary Table 2. List of contaminants, clusters and their level of identification based on the Metabolomics Standards Initiative (MSI).

Full name	Abbreviation	Cluster	Level of identification
Bisphenol S	BPS	CC1	Level 1
Deoxynivalenol	DON	CC1	Level 1
Monobutyl phthalate	MBP	CC1	Level 2
Perfluorodecanoic acid	PFDA	CC1	Level 1
Methylperfluorooctane sulfonamidoacetic acid	MeFOSAA	CC1	Level 2
Environmental Contaminant 1	EC1	CC1	Level 2
Environmental Contaminant 2	EC2	CC1	Level 2
a-Zearalanol	ZEL	CC1	Level 1
Ethylparaben	EP	CC2	Level 1
Methylparaben	MP	CC2	Level 1
Propylparaben	PP	CC2	Level 1
Perfluorohexanesulfonic acid Branched	PFHxSBr	CC3	Level 1
Perfluorohexanesulfonic acid	PFHxS	CC3	Level 1
Perfluorooctanoic acid Branched 1	PFOABr1	CC4	Level 1
Perfluorooctanoic acid Linear 1	PFOAL1	CC4	Level 1
Perfluorooctanoic acid Branched 2	PFOABr2	CC4	Level 1
Perfluorooctanoic acid Linear 2	PFOAL2	CC4	Level 1
Perfluorooctanesulfonic acid Branched	PFOSBr	CC4	Level 1
Perfluorooctanesulfonic acid	PFOS	CC4	Level 1
Environmental Contaminant 3	EC3	CC4	Level 2

Supplementary Table 3. Two-way analysis of variance (ANOVA) for cord serum lipids (cluster LC1 to LC8) impacted by contaminants exposure (Contaminant cluster CC1 to CC4). The samples were grouped based on contaminants exposure quartiles (1 to 4) and Groups (Control/Cases). The statistical significance levels are represented by p-values ($p < 0.05$ marked bold, p-values < 0.1 in italics).

Contaminant cluster	Lipid cluster	Factor 1: Contaminant quartiles	Factor 2: Groups (Control, Cases)	Interactions F1*F2
CC1	LC1	0.291	0.203	0.467
CC1	LC2	0.271	0.674	0.568
CC1	LC3	**9.59×10^{-4}**	0.884	0.701
CC1	LC4	**6.95×10^{-4}**	0.983	0.850
CC1	LC5	0.519	*0.055*	*0.064*
CC1	LC6	0.265	0.124	0.111
CC1	LC7	**2.03×10^{-2}**	0.919	0.936
CC1	LC8	0.869	0.449	0.260
CC2	LC1	0.706	0.195	0.895
CC2	LC2	0.683	0.719	0.471
CC2	LC3	0.814	0.901	0.992
CC2	LC4	0.531	0.973	0.958
CC2	LC5	0.995	*0.071*	0.947
CC2	LC6	0.731	0.176	0.608
CC2	LC7	*0.096*	0.960	0.139
CC2	LC8	0.298	0.378	0.917
CC3	LC1	0.578	0.220	0.721
CC3	LC2	0.283	0.803	0.856
CC3	LC3	0.276	0.981	0.730
CC3	LC4	0.581	0.840	0.747
CC3	LC5	**1.43×10^{-4}**	*0.061*	0.433
CC3	LC6	**3.27×10^{-3}**	0.194	0.329
CC3	LC7	0.909	0.967	0.160
CC3	LC8	0.179	0.455	0.919
CC4	LC1	0.249	0.141	*0.074*
CC4	LC2	0.246	0.534	0.416
CC4	LC3	0.194	0.713	**0.027**
CC4	LC4	0.221	0.868	**0.032**
CC4	LC5	**0.019**	**0.026**	*0.068*
CC4	LC6	0.361	0.123	0.348
CC4	LC7	0.172	0.774	0.521
CC4	LC8	*0.095*	0.296	0.523

Supplementary Table 4. Post-Hoc test that followed a two-way analysis of variance for cord serum lipids (cluster LC1 to LC8) affected by contaminant exposure (Contaminant cluster CC1 to CC4). The Post-Hoc Tukeys' HSD test was used for pairwise comparison between metabolite levels (along quartiles). The statistical significance levels are indicated by p-values, with values less than 0.05 marked in bold and values less than 0.1 in italics.

Contaminant cluster	Lipid cluster	Factor 1: Contaminant quartiles					
		Q2-Q1	Q3-Q1	Q4-Q1	Q3-Q2	Q4-Q2	Q4-Q3
CC1	LC1	1.000	0.379	0.762	0.371	0.755	0.925
CC1	LC2	0.407	0.820	0.271	0.905	0.994	0.785
CC1	LC3	0.317	0.106	**$3.68{\times}10^{-4}$**	0.942	*0.091*	0.289
CC1	LC4	0.166	*0.081*	**$2.38{\times}10^{-4}$**	0.988	0.156	0.292
CC1	LC5	0.991	0.904	0.866	0.980	0.711	0.467
CC1	LC6	0.216	0.659	0.911	0.864	0.585	0.962
CC1	LC7	0.759	0.154	**0.018**	0.675	0.204	0.837
CC1	LC8	0.988	0.870	0.915	0.971	0.987	0.999
CC2	LC1	0.929	0.952	0.992	0.665	0.988	0.849
CC2	LC2	0.957	0.633	0.864	0.903	0.993	0.977
CC2	LC3	0.986	0.935	0.992	0.788	0.926	0.990
CC2	LC4	0.993	0.694	0.943	0.523	0.839	0.952
CC2	LC5	1.000	0.998	0.998	0.997	0.997	1.000
CC2	LC6	0.968	0.798	0.745	0.968	0.946	1.000
CC2	LC7	0.148	0.989	0.325	0.277	0.975	0.516
CC2	LC8	0.995	0.341	0.640	0.483	0.786	0.961
CC3	LC1	0.609	0.625	0.862	1.000	0.971	0.975
CC3	LC2	0.807	0.220	0.914	0.731	0.995	0.585
CC3	LC3	0.870	0.943	0.729	0.997	0.277	0.384
CC3	LC4	1.000	0.985	0.756	0.977	0.787	0.539
CC3	LC5	0.216	0.343	**$4.93{\times}10^{-5}$**	0.994	**0.048**	**0.024**
CC3	LC6	0.490	0.273	**0.001**	0.979	0.101	0.231
CC3	LC7	0.999	0.983	0.986	0.953	0.998	0.893
CC3	LC8	0.299	0.702	0.174	0.910	0.991	0.772
CC4	LC1	0.969	0.655	0.629	0.376	0.353	1.000
CC4	LC2	0.759	0.826	0.178	0.999	0.718	0.646
CC4	LC3	0.863	0.462	0.167	0.903	0.573	0.932
CC4	LC4	0.812	0.727	0.157	0.999	0.620	0.718
CC4	LC5	0.984	0.215	0.139	0.101	*0.059*	0.996
CC4	LC6	0.999	0.881	0.470	0.818	0.389	0.895
CC4	LC7	0.993	0.504	0.449	0.345	0.300	1.000
CC4	LC8	0.928	0.754	0.315	0.379	*0.093*	0.886

Supplementary Table 5. Two-way analysis of variance (ANOVA) for cord serum polar metabolites (cluster PC1 to PC12) impacted by contaminants exposure (Contaminant cluster CC1 to CC4). The samples were grouped based on contaminants exposure quartiles (1 to 4) and Groups (Control/Cases). The statistical significance levels are represented by p-values ($p < 0.05$ marked bold, p-values < 0.1 in italics).

Contaminant cluster	Polar metabolite cluster	Factor 1: Contaminant quartiles	Factor 2: Groups (Control, Cases)	Interactions F1*F2
CC1	PC1	**$<2\times10^{-16}$**	0.685	0.331
CC1	PC2	**$<2\times10^{-16}$**	**0.022**	0.516
CC1	PC3	0.780	0.237	0.380
CC1	PC4	**3.11×10^{-11}**	**0.001**	0.876
CC1	PC5	**0.026**	0.554	0.401
CC1	PC6	**$<2\times10^{-16}$**	0.338	0.378
CC1	PC7	**0.017**	0.726	0.533
CC1	PC8	***0.082***	0.963	0.533
CC1	PC9	***0.085***	0.703	0.824
CC1	PC10	**1.23×10^{-11}**	0.967	0.731
CC1	PC11	**4.09×10^{-9}**	**3.98×10^{-4}**	0.544
CC1	PC12	***0.068***	0.356	0.824
CC2	PC1	**0.025**	0.574	0.186
CC2	PC2	**1.89×10^{-4}**	***0.097***	0.817
CC2	PC3	0.836	0.250	0.723
CC2	PC4	**0.021**	**0.004**	0.637
CC2	PC5	0.338	0.498	0.789
CC2	PC6	**0.001**	0.236	0.446
CC2	PC7	**0.006**	0.735	0.122
CC2	PC8	**0.006**	0.631	***0.091***
CC2	PC9	0.718	0.577	0.250
CC2	PC10	***0.051***	0.727	0.567
CC2	PC11	0.469	**0.004**	0.793
CC2	PC12	0.162	0.310	**0.011**
CC3	PC1	**2.02×10^{-6}**	0.589	0.829
CC3	PC2	0.131	0.199	0.550
CC3	PC3	0.115	0.259	0.665
CC3	PC4	**0.044**	**0.011**	0.515
CC3	PC5	***0.095***	0.538	0.396
CC3	PC6	**0.017**	0.160	0.273
CC3	PC7	0.509	0.738	**0.004**
CC3	PC8	**0.002**	0.809	0.628
CC3	PC9	0.905	0.591	0.232
CC3	PC10	***0.094***	0.554	0.953
CC3	PC11	**0.044**	**0.005**	0.384
CC3	PC12	0.822	0.318	0.148
CC4	PC1	0.242	0.468	0.861

CC4	PC2	0.376	0.118	0.678
CC4	PC3	**0.034**	0.143	0.855
CC4	PC4	0.138	**0.002**	0.666
CC4	PC5	**0.027**	0.273	0.473
CC4	PC6	**0.001**	0.398	0.617
CC4	PC7	0.686	0.833	**0.032**
CC4	PC8	**0.010**	0.427	***0.090***
CC4	PC9	0.659	0.738	0.525
CC4	PC10	**0.019**	0.621	0.943
CC4	PC11	0.183	**0.002**	0.677
CC4	PC12	0.579	0.401	**0.006**

Supplementary Table 6. Post-Hoc test that followed a two-way analysis of variance for cord serum polar metabolites (cluster PC1 to PC12) affected by contaminant exposure (Contaminant cluster CC1 to CC4). The Post-Hoc Tukeys' HSD test was used for pairwise comparison between metabolite levels (along quartiles). The statistical significance levels are indicated by p-values, with values less than 0.05 marked in bold and values less than 0.1 in italics.

Contaminant cluster	Polar metabolite cluster	Factor 1: Contaminant quartiles					
		Q2-Q1	Q3-Q1	Q4-Q1	Q3-Q2	Q4-Q2	Q4-Q3
CC1	PC1	**0.002**	**10^{-8}**	**10^{-8}**	**0.021**	**10^{-8}**	**0.006**
CC1	PC2	10^{-8}	**10^{-8}**	**10^{-8}**	0.813	**10^{-8}**	**0.004**
CC1	PC3	1.000	0.862	0.998	0.875	0.997	0.773
CC1	PC4	**4×10^{-7}**	**1.84×10^{-4}**	**10^{-8}**	0.571	0.357	**0.020**
CC1	PC5	0.494	0.653	**0.014**	0.995	0.362	0.242
CC1	PC6	**4.76×10^{-10}**	**9.10×10^{-13}**	**8.78×10^{-13}**	**9.06×10^{-4}**	**5.71×10^{-7}**	0.362
CC1	PC7	0.539	0.853	**0.011**	0.951	0.288	0.100
CC1	PC8	0.139	0.321	***0.096***	0.972	0.998	0.930
CC1	PC9	1.000	0.603	0.533	0.665	0.471	***0.051***
CC1	PC10	**2.4×10^{-6}**	**7×10^{-7}**	**10^{-8}**	0.993	0.155	0.262
CC1	PC11	**1.71×10^{-5}**	**1.93×10^{-4}**	**10^{-8}**	0.950	0.346	0.127
CC1	PC12	0.922	0.918	0.218	0.581	0.564	***0.052***
CC2	PC1	**0.072**	**0.035**	0.690	0.992	0.543	0.375
CC2	PC2	0.998	0.938	**0.003**	0.869	**0.006**	**3.40×10^{-4}**
CC2	PC3	0.978	0.798	0.988	0.957	1.000	0.936
CC2	PC4	0.707	0.974	**0.020**	0.918	0.252	***0.064***
CC2	PC5	0.615	0.288	0.877	0.944	0.966	0.737
CC2	PC6	***0.066***	***0.075***	0.913	1.000	**0.011**	**0.012**
CC2	PC7	0.812	0.522	0.158	0.113	0.621	**0.004**
CC2	PC8	0.116	**0.013**	**0.011**	0.844	0.814	1.000
CC2	PC9	0.988	0.990	0.851	0.923	0.963	0.685
CC2	PC10	0.721	0.669	0.548	1.000	***0.085***	***0.070***
CC2	PC11	0.806	0.501	0.998	0.958	0.886	0.610
CC2	PC12	0.883	0.898	0.463	0.481	0.887	0.140
CC3	PC1	0.967	**0.002**	**5.92×10^{-5}**	**0.008**	**4.08×10^{-4}**	0.850
CC3	PC2	0.125	0.585	0.240	0.790	0.989	0.929
CC3	PC3	0.734	0.495	***0.076***	0.981	0.506	0.748
CC3	PC4	0.473	0.509	0.983	**0.026**	0.272	0.741
CC3	PC5	0.924	0.109	0.292	0.359	0.664	0.960
CC3	PC6	0.633	0.325	**0.009**	0.956	0.199	0.464
CC3	PC7	0.987	0.919	0.721	0.764	0.512	0.977
CC3	PC8	0.820	**0.030**	**0.005**	0.229	***0.065***	0.940
CC3	PC9	0.994	0.996	0.886	1.000	0.965	0.957
CC3	PC10	0.114	0.619	0.149	0.737	0.999	0.803
CC3	PC11	0.601	0.443	0.866	**0.034**	0.185	0.891

CC3	PC12	0.997	0.800	0.986	0.893	0.999	0.943
CC4	PC1	0.479	0.196	0.713	0.945	0.983	0.794
CC4	PC2	0.988	0.939	0.710	0.994	0.508	0.359
CC4	PC3	0.597	0.854	0.383	0.174	**0.025**	0.859
CC4	PC4	0.994	0.975	0.229	0.908	0.139	0.453
CC4	PC5	0.981	0.146	***0.060***	0.302	0.147	0.982
CC4	PC6	0.806	1.000	**0.019**	0.820	**0.001**	**0.018**
CC4	PC7	1.000	0.761	0.934	0.726	0.914	0.980
CC4	PC8	0.637	***0.065***	**0.011**	0.565	0.217	0.922
CC4	PC9	0.997	1.000	0.678	0.999	0.801	0.730
CC4	PC10	0.976	**0.036**	1.000	0.102	0.972	**0.035**
CC4	PC11	0.918	0.716	0.738	0.975	0.347	0.165
CC4	PC12	0.925	0.998	0.867	0.859	0.505	0.931

Supplementary Table 7. provides information on the pathways identified through pathway enrichment analysis using MFN pathway maps for controls. It includes the pathways, their corresponding p-values, and the number of metabolites in each pathway, including the total size, hits, and significant hits. The combined p-value was calculated by combining GSEA and Mummichog scores. This table lists only those pathways that have a combined p-value of less than 0.05.

Name of the pathways	Total size	Hits	Significant hits	Mummichog P values	GSEA P values	Combined P values
Tyrosine metabolism	160	50	40	0.0207	0.0099	0.00195
Tryptophan metabolism	94	31	26	0.02223	0.0198	0.00384
Valine, leucine and isoleucine degradation	65	12	11	0.049	0.0101	0.00426
Pyrimidine metabolism	70	20	17	0.05297	0.0099	0.00449
Urea cycle/amino group metabolism	85	26	21	0.07829	0.01	0.00638
Glycine, serine, alanine and threonine metabolism	88	27	21	0.1365	0.01	0.01037
Butanoate metabolism	34	11	10	0.06911	0.02083	0.01086
Beta-Alanine metabolism	20	9	8	0.1347	0.01099	0.01112
Caffeine metabolism	11	4	4	0.1909	0.01176	0.01594
Fructose and mannose metabolism	33	7	7	0.05475	0.04301	0.01661
Sialic acid metabolism	107	14	11	0.2462	0.01	0.01725
Aminosugars metabolism	69	10	8	0.286	0.01064	0.02068
Propanoate metabolism	31	9	8	0.1347	0.03297	0.02849
Pyruvate Metabolism	20	11	8	0.4575	0.01099	0.03164
Hexose phosphorylation	20	10	7	0.5454	0.01099	0.03667
Methionine and cysteine metabolism	94	17	14	0.1189	0.05941	0.04206
Glutamate metabolism	15	9	8	0.1347	0.05495	0.04371
Selenoamino acid metabolism	35	6	5	0.3404	0.02222	0.04451
Histidine metabolism	33	10	9	0.09685	0.08511	0.0478
Glycosphingolipid metabolism	67	20	15	0.2781	0.0297	0.04787

Supplementary Table 8. provides information on the pathways identified through pathway enrichment analysis using KEGG pathway maps for controls. It includes the pathways, their corresponding p-values, and the number of metabolites in each pathway, including the total size, hits, and significant hits. The combined p-value was calculated by combining GSEA and Mummichog scores. This table lists only those pathways that have a combined p-value of less than 0.05.

**Name of the pathways**	**Total size**	**Hits**	**Significant hits**	**Mummichog P values**	**GSEA P values**	**Combined P values**
Aminoacyl-tRNA biosynthesis	22	14	14	0.0072	0.0101	0.00077
Glycine, serine and threonine metabolism	30	14	14	0.0072	0.0101	0.00077
Tyrosine metabolism	42	21	19	0.02887	0.0099	0.00262
Fructose and mannose metabolism	20	6	6	0.1235	0.01053	0.00994
Valine, leucine and isoleucine biosynthesis	8	6	6	0.1235	0.01053	0.00994
Valine, leucine and isoleucine degradation	35	10	9	0.158	0.0101	0.01188
Phenylalanine metabolism	10	8	7	0.2676	0.0101	0.01869
Phenylalanine, tyrosine and tryptophan biosynthesis	4	3	3	0.3529	0.01333	0.02991
Amino sugar and nucleotide sugar metabolism	35	9	6	0.7479	0.0101	0.04446

Supplementary Table 9. provides information on the pathways identified through pathway enrichment analysis using MFN pathway maps for cases. It includes the pathways, their corresponding p-values, and the number of metabolites in each pathway, including the total size, hits, and significant hits. The combined p-value was calculated by combining GSEA and Mummichog scores. This table lists only those pathways that have a combined p-value of less than 0.05.

**Name of the pathways**	**Total size**	**Hits**	**Significant hits**	**Mummichog P values**	**GSEA P values**	**Combined P values**
Urea cycle/amino group metabolism	85	21	8	0.04279	0.01053	0.00392
Beta-Alanine metabolism	20	9	5	0.01973	0.02469	0.0042
Glutamate metabolism	15	8	4	0.05732	0.0125	0.0059
Glutathione Metabolism	19	4	3	0.02771	0.02817	0.00637
Aminosugars metabolism	69	10	5	0.03316	0.02469	0.00664
Valine, leucine and isoleucine degradation	65	11	5	0.05113	0.02326	0.0092
Alanine and Aspartate Metabolism	30	10	4	0.1235	0.0125	0.01154
Pyruvate Metabolism	20	10	4	0.1235	0.0125	0.01154
Glycerophospholipid metabolism	156	21	7	0.1102	0.02105	0.01639
Glycine, serine, alanine and threonine metabolism	88	25	7	0.2259	0.01064	0.0169
Butanoate metabolism	34	10	4	0.1235	0.02299	0.01949
Carbon fixation	10	2	2	0.04075	0.09524	0.02543
Aspartate and asparagine metabolism	114	30	6	0.591	0.01087	0.03885
Pyrimidine metabolism	70	18	6	0.1357	0.05435	0.04358
Arginine and Proline Metabolism	45	19	5	0.3361	0.02247	0.04445

Supplementary Table 10. provides information on the pathways identified through pathway enrichment analysis using KEGG pathway maps for cases. It includes the pathways, their corresponding p-values, and the number of metabolites in each pathway, including the total size, hits, and significant hits. The combined p-value was calculated by combining GSEA and Mummichog scores. This table lists only those pathways that have a combined p-value of less than 0.05.

Name of the pathways	Total size	Hits	Significant hits	Mummichog P values	GSEA P values	Combined P values
Aminoacyl-tRNA biosynthesis	22	15	7	0.03758	0.01099	0.00363
Glycine, serine and threonine metabolism	30	14	6	0.08186	0.01124	0.00735
Phosphonate and phosphinate metabolism	4	2	2	0.05452	0.03226	0.01291
Alanine, aspartate and glutamate metabolism	28	12	5	0.1234	0.03448	0.02749
Glyoxylate and dicarboxylate metabolism	31	7	3	0.2106	0.02299	0.03066
Pantothenate and CoA biosynthesis	17	5	3	0.0864	0.05882	0.03193
Glycolysis or Gluconeogenesis	23	3	2	0.1385	0.04286	0.03637
Arginine and proline metabolism	37	19	4	0.6919	0.01099	0.0447
